# Advancing the Study of Mindfulness-Based Interventions in Relation to Psychological Health

**DOI:** 10.3390/ijerph20085473

**Published:** 2023-04-11

**Authors:** Rachel G. Lucas-Thompson, Megan J. Moran

**Affiliations:** Department of Human Development & Family Studies, Colorado State University, 1570 Campus Delivery, Fort Collins, CO 80523-1570, USA; megan.joy.moran@colostate.edu

## 1. Introduction

Since 2006, there has been exponential growth in the number of publications on mindfulness [1]. Recently conducted meta-analyses support the benefits of mindfulness-based interventions (MBIs) for diverse elements of psychological health, with benefits evident in both clinical and non-clinical samples [2,3,4,5,6,7,8,9,10,11]. The evidence base supporting the implementation and dissemination of MBIs is growing, but remains limited in critical ways, in part because wide-spread dissemination preceded a solid empirical evidence base [12]. Notably, much of the evidence evaluating the benefits of MBIs for psychological health focuses on relatively short-term changes in health, in samples of primarily white adults from Western countries, with a reliance on designs that lack an active control comparison. In order to advance the study of MBIs in relation to psychological health, it is necessary to conduct studies that are process-oriented, test mediators and/or moderators of intervention effects, evaluate the durability of effects, compare the effects of MBIs to other active intervention conditions; and innovate in both the delivery and assessment of MBIs.

## 2. Process-Oriented Investigations

A key priority for the field is increasing the scientific understanding of the mechanisms via which MBIs influence health outcomes. The proposed mediators of MBIs vary across multiple theoretical models; however, some of the most well-supported mediators include attentional processes (e.g., mindful attention), self-regulation (e.g., emotion regulation), and self-related processes (e.g., rumination) [13]. There is also a growing interest in understanding the ways by which MBIs operate at a neurobiological level. Indeed, evidence suggests that numerous brain regions are affected by MBIs and that they may also drive subsequent improvements in outcomes of interest [14,15]. Perhaps because the field lacks a unified theory of MBIs, a wide net has been cast for potential mediators. To strengthen our confidence in mediators of MBIs, there is a particular need for studies that replicate prior mediation findings, utilizing designs that include assessments across multiple timepoints to robustly test causality and the temporal ordering of changes. 

Research that includes thoughtful and rigorous tests by theoretically informed moderators is also needed. Individual characteristics (e.g., age, personality traits, meditation experience) moderate MBIs in some cases, as does intervention type (e.g., universal, indicated); however, our understanding of the moderators of MBIs remains underdeveloped [8,16,17]. In terms of sociodemographic characteristics, tests of moderation that can accommodate the intersectional nature of identity would be particularly valuable. Evidence also suggests that fidelity-related factors and intervention context may moderate MBs; however, these moderators are often not captured, or are assessed with single indicators (e.g., attendance) [18]. Studies of MBIs that prioritize better understanding these factors would support the continued refinement of theory and provide important direction in terms of individuals who may benefit most, as well as opportunities for optimizing implementation. 

## 3. An Emphasis on Long-Term Change

Many investigations of MBIs base their conclusions on the changes from before to immediately after program cessation. For instance, in most of the meta-analytic investigations cited above, the percentage of studies included that examined durability of effects at a follow-up ranged was approximately 20–30% [5,7,8,10,11]. At most, 53% of studies presented data about effects at a follow-up period [11]. In addition, even when studies evaluated the extent to which benefits persist for some period beyond program cessation, most studies have had relatively short periods of follow-up, with most focusing on durability across a span of time ranging from only several weeks to less than 6 months [7,8,9,10,11]. Critically, much of this evidence suggests that the effects of MBIs are durable, such that effects at post-test are maintained over these follow-up periods [9,11] In addition, direct tests of the extent to which the length of the follow-up period moderates program effects suggest that length does not influence these effects [7]. Therefore, this early evidence is quite encouraging about the possible long-term durability of the benefits of MBIs for psychological health. However, a critical next step is to understand durability over longer periods of time, the extent to which these long-term benefits are evident in relation to active controls, and the mechanisms of and individual differences in long-term program effects. 

## 4. The Importance of Active Controls

A frequently noted weakness of MBIs is their lack of active controls [11,19]. Many evaluations of MBIs rely on single-arm pre–post designs [19], which are vulnerable to numerous threats to internal validity; most other evaluations rely on passive controls [3,5,6,7,19], which control for changes due to history or time, but little else. It is critical to compare the effects of MBIs to other active controls to account for changes due to demand characteristics, placebo effects, and non-specific intervention effects (e.g., attention from a trained facilitator, connections with other participants). Importantly, there is much stronger evidence for the benefits of MBIs based on studies utilizing a single-arm or passive control group. For instance, many positive effects of MBIs that are evident in studies with weaker designs are no longer evident when effects are restricted to those based on comparison with an active control [4]. Direct tests of the extent to which MBIs are more beneficial than different types of control conditions suggest that, although they are most effective than minimal treatment, non-specific, and specific active controls, they are not more effective than “first-line evidence-based therapies” like cognitive-behavioral therapy [20]. However, some meta-analyses have found no evidence of systematic differences in the effect sizes between active and passive control designs [5]. Unfortunately, change has moved slowly in this domain of the study of mindfulness-based interventions. Calls to increase the use of active controls have been made for over 20 years [21], but there have been no significant increases in the use of active control conditions over the last several decades [19], perhaps in part because these studies are more likely to have null effects and therefore suffer from the file-drawer problem. It is critical to better understand the extent to which MBIs are effective beyond possible effects of history, time, placebo effects, demand characteristics, and non-specific intervention effects to inform their dissemination and implementation as well as a process-oriented understanding of the specific mechanisms by which MBIs operate. 

## 5. Novel Delivery Methods

As with the proliferation of the study of mindfulness, investigations of mobile health (mHealth) have increased since 2006 [22]. Currently, there are hundreds of extant publicly available cellphone applications for practicing mindfulness. However, the majority of these are low-quality, and lack rigorous or reproducible evidence in support of their effectiveness [23]. At the same time, meta-analyses have indicated that, relative to control interventions, “self-help” MBIs significantly increase mindfulness and decrease mental health symptoms [24]. But, particularly in some sub-groups of participants, rates of compliance are problematically low with key elements of mindfulness-based interventions responsible for transferring the intervention content into daily life, such as regular home practice [25]. As a result, a critical next step in the science and implementation of MBIs is incorporating and evaluating mHealth approaches that hold promise for boosting intervention effects, broadening intervention reach, and improving skill transfer [26]. 

## 6. Innovations in Measurement and Analysis

Research into MBIs is dominated by pre–post designs and retrospective self-report assessment. These methodological limitations reduce our ability to identify mechanisms, to understand durability of results, and, in some cases, to gain an accurate estimate of how MBIs may be affecting mindfulness. Indeed, meta-analytic evidence suggests that about 50% of MBIs do not lead to increases in self-reported mindfulness [27]. Studies with multiple assessment timepoints are better suited for identifying underlying mechanisms of MBIs. Additionally, experience sampling methods, in particular, hold promise for capturing more reliable self-reports of mindfulness [28]. Also needed is research that focuses on refining existing self-report measures of mindfulness through rigorous psychometric analysis, as well as on developing and testing novel supplements to self-reporting. These should include, but not be limited tom qualitative methods, behavioral tasks, and biological and neuroscientific measures [29,30]. 

## 7. Concerns about Generalizability: A Focus on Underrepresented Populations

There is a need for increased diversity in research on MBIs. Globally, research on MBIs has primarily been conducted with subjects living in western, industrialized, rich, and democratic countries. In the United States, specifically, the overwhelming majority of participants in studies of MBIs are white and generally more highly educated than the general population [31]. Thus, if research on MBIs is to advance health equity, the field should prioritize advancing the understanding of how MBIs can support society’s most vulnerable individuals. A first step toward that goal is the recruitment of more diverse samples. The development of a lifespan perspective is also important. There has been significant growth in research into MBIs in children and adolescents [4,7], and continued focus on the earlier part of the lifespan will be particularly important for understanding the ways in which the end of the lifespan has been neglected; thus, there is a particular need for research into MBIs in older adults [11].

## 8. Conclusions

It is critical that the evidence base evaluating the benefits of MBIs not only continues to grow, but that also that improvements are made in a variety of key ways outlined and reviewed here. Academics, scholars and researchers are invited to submit studies to this Special Issue of *IJERPH*, entitled “Psychological Health and Benefits of Mindfulness-Based Interventions’, that investigate any of these areas for growth in order to advance the study of MBIs.
